# From Dysbiosis to Distress: The Gut–Brain Connection in Trauma-Related Disorders

**DOI:** 10.3390/nu18030530

**Published:** 2026-02-05

**Authors:** Giuseppe Marano, Luca Lo Giudice, Elettra Specogna, Luca Chisari, Caterina Brisi, Gianandrea Traversi, Osvaldo Mazza, Marianna Mazza

**Affiliations:** 1 Department of Neuroscience, Head-Neck and Chest, Section of Psychiatry, Fondazione Policlinico Universi-Tario Agostino Gemelli IRCCS, Largo Agostino Gemelli 8, 00168 Rome, Italy; 2 Department of Neuroscience, Section of Psychiatry, Università Cattolica del Sacro Cuore, 00168 Rome, Italy; 3 Unit of Medical Genetics, Department of Laboratory Medicine, Ospedale Isola Tiberina-Gemelli Isola, 00186 Rome, Italy; gianandrea.traversi@gmail.com; 4 Spine Surgery Department, Bambino Gesù Children’s Hospital IRCCS, 00168 Rome, Italy; osvaldo.mazza1973@hotmail.it

**Keywords:** microbiota, gut–brain axis, PTSD, trauma, dysbiosis, neuroinflammation, psychobiotics, HPA axis, mental health

## Abstract

**Background**: Post-traumatic stress disorder (PTSD) and trauma-related disorders are increasingly recognized as complex conditions involving not only psychological and neurobiological factors but also systemic physiological alterations. Among these, growing evidence points to the crucial role of the gut–brain axis in modulating stress responses, emotional regulation, and neuroinflammation. **Objective**: This narrative review aims to synthesize the emerging literature on the interactions between the gut microbiota and the central nervous system in PTSD and trauma-related disorders, highlighting potential mechanisms and therapeutic implications. **Methods**: A comprehensive search of the literature was conducted across PubMed and Scopus and Web of Science as primary bibliographic databases, focusing on clinical, preclinical, and translational studies published in the last two decades. Emphasis was placed on studies exploring the microbiota’s influence on neuroendocrine and immune pathways relevant to trauma, as well as intervention studies targeting the gut–brain axis. **Results**: Evidence suggests that dysbiosis and gut permeability alterations are associated with PTSD symptomatology, possibly via mechanisms involving hypothalamic–pituitary–adrenal (HPA) axis dysregulation, neuroinflammation, vagal signaling, and altered neurotransmitter production. Preclinical models support a bidirectional relationship between early-life stress, trauma, and gut microbiota alterations. Furthermore, preliminary clinical data indicate that interventions such as probiotics, diet modulation, and psychobiotics may exert beneficial effects on trauma-related psychopathology. **Conclusions**: The gut–brain axis represents a promising framework for understanding the pathophysiology of PTSD and related conditions. Although research is still in its early stages, targeting gut microbiota may offer novel preventive and therapeutic strategies. Further longitudinal and mechanistic studies are needed to validate these findings and guide clinical translation.

## 1. Introduction

### 1.1. Definitions: PTSD and Trauma-Related Disorders

Post-traumatic stress disorder (PTSD) is a trauma- and stressor-related psychiatric condition that emerges following exposure to life-threatening or highly distressing events and is characterized by intrusive recollections, avoidance behaviors, hyperarousal, and persistent negative alterations in mood and cognition [1,2,3]. Beyond its core psychological symptoms, PTSD is increasingly conceptualized as a systemic disorder involving widespread neurobiological dysregulation across endocrine, immune, and metabolic systems [1,4].

At the neuroendocrine level, PTSD is associated with the distinctive dysregulation of the hypothalamic–pituitary–adrenal (HPA) axis, characterized by reduced basal cortisol levels alongside enhanced corticotropin-releasing hormone (CRH) signaling, reflecting a paradoxical state of hypocortisolism despite chronic stress exposure [2,5]. This altered stress physiology has been consistently linked to long-term maladaptive stress responses and impaired negative feedback regulation [4].

Such neuroendocrine imbalance contributes to sustained immune activation, which is consistently associated with PTSD and may play a contributory role in maintaining inflammatory and neuroendocrine dysregulation. Individuals with PTSD frequently exhibit elevated circulating pro-inflammatory mediators, including interleukin-6 (IL-6), tumor necrosis factor-α (TNF-α), and C-reactive protein (CRP), which may persist for years after trauma exposure and promote a chronic low-grade inflammatory state [1,3,6]. This inflammatory phenotype is increasingly implicated in the somatic comorbidities commonly observed in PTSD, including cardiovascular disease, metabolic dysregulation, and gastrointestinal disorders [3,4].

Taken together, PTSD represents a prototypical stress-related condition in which psychological trauma translates into enduring biological alterations, bridging psychiatry, psychoneuroendocrinology, and immunology [2,4,7].

Trauma exposure can be followed by a heterogeneous range of psychiatric outcomes. PTSD represents one specific post-traumatic phenotype characterized by fear-based re-experiencing, avoidance, negative changes in cognition and mood, and hyperarousal, typically persisting beyond the acute post-event period. However, other trauma- and stressor-related disorders may present after adversity with partially overlapping but conceptually distinct symptom constellations and trajectories (e.g., acute stress disorder in the first month; adjustment disorder in response to non-traumatic stressors; prolonged grief disorder following bereavement). Neuroimaging findings in PTSD should be interpreted against the broader landscape of stress-related psychopathology, where partially overlapping fronto-limbic abnormalities are common [8]. Harnett and colleagues synthesize evidence that PTSD is most consistently characterized by dysfunction across a fear learning and memory network centered on the amygdala, hippocampus, and prefrontal/cingulate regions, spanning abnormalities in brain function, structure, and biochemistry. Importantly, several of these alterations (e.g., perturbations in prefrontal–limbic regulation and stress-related neurochemical systems) are also reported in major depressive disorder and anxiety disorders, suggesting that single-region findings are unlikely to be diagnostically specific and may instead index transdiagnostic effects of chronic stress. In this context, PTSD specificity is best framed as a pattern involving disrupted threat learning/extinction and regulatory control within this circuit, rather than as a unique biomarker in isolation. Finally, adjustment disorder remains comparatively under-represented in the neuroimaging literature, limiting confident statements about disorder-specific brain signatures relative to PTSD [8].

To avoid conceptual conflation, the present work focuses explicitly on PTSD and does not treat findings as interchangeable with other trauma- and stressor-related diagnoses.

### 1.2. The Gut–Brain Axis: A Brief Primer

The gut–brain axis (GBA) is a complex bidirectional communication network linking the gastrointestinal tract and the central nervous system through neural, immune, endocrine, and microbial-metabolite pathways [9,10,11,12]. Central to this system is the intestinal microbiota, which regulates metabolic processes, modulates mucosal and systemic immune responses, and communicates with the brain via the vagus nerve and enteric nervous system [13,14]. Clinical and experimental evidence across disciplines supports the gut microbiota–brain axis as a conserved regulatory system influencing cognition, emotion, and stress physiology [15].

Under physiological conditions, commensal microorganisms support intestinal homeostasis by downregulating epithelial inflammatory responses, promoting mucus production, stimulating antimicrobial peptide expression, and facilitating epithelial repair following injury [16,17]. Microbiota composition is highly dynamic and influenced by multiple factors, including age, diet, geographic environment, air pollution, medication exposure, infections, toxins, and host genetic background [18,19,20,21].

Exposure to chronic psychological stress disrupts this finely tuned ecosystem. Stress-related mediators such as cortisol and catecholamines can increase intestinal permeability and alter microbial diversity, facilitating the translocation of bacterial components such as lipopolysaccharides (LPS) into the systemic circulation [5,9,14]. These processes amplify peripheral inflammation and promote microglial activation, thereby influencing emotional regulation and stress responsivity [7,13].

Conversely, microbial-derived metabolites, particularly short-chain fatty acids (SCFAs), exert neuroactive and immunomodulatory effects, influencing neurotransmission, neuroinflammation, and HPA axis regulation [22,23,24]. These findings underscore the central role of the gut microbiome in stress resilience and emotional homeostasis [13,25].

Collectively, the GBA represents a dynamic homeostatic network in which the microbiota functions both as a target of stress-induced perturbations and as an active mediator of stress-related biological responses [9,10,14].

### 1.3. Scope and Rationale of the Review

From a clinical psychiatry perspective, growing evidence supports the inclusion of gut microbiota biology within psychiatric nosology and mechanistic reasoning [26]. The concept of “mind-altering microorganisms” has provided a theoretical foundation for understanding how microbial communities may shape cognition, emotion, and stress reactivity [27]. Accumulating evidence implicates gut microbiota dysbiosis and intestinal barrier dysfunction in the development and persistence of stress-related psychopathology [9,14,23]. PTSD offers a particularly informative clinical model to investigate these interactions, as both human and animal studies demonstrate trauma-associated alterations in microbial composition, increased gut permeability, and systemic inflammatory activation [23,28,29].

These observations have stimulated growing interest in the gut–brain–microbiota axis as a potential therapeutic target. Emerging interventions, including probiotic supplementation, dietary modulation, and lifestyle-based strategies, have shown preliminary efficacy in modulating stress responses and PTSD-related symptoms [22,30,31].

**Figure 1:**
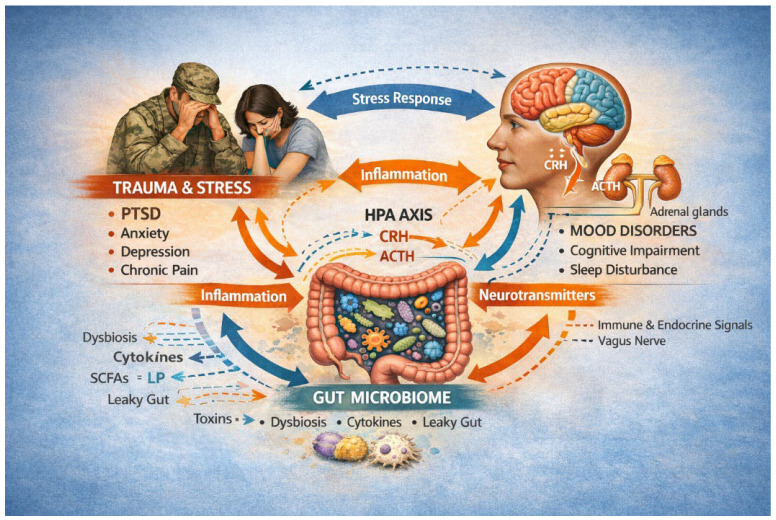
Conceptual model of Gut–Brain-Trauma Pathways. HPA: Hypothalamic–Pituitary–Adrenal axis; CRH: Corticotropin-Releasing Hormone; SCFAs = Short-Chain Fatty Acids; LP = Lipopolysaccharide; PTSD = Post-Traumatic Stress Disorder. Solid arrows indicate primarily causal relationship supported by preclinical evidence; dashed arrows represent associative or bidirectional relationships in clinical studies.

However, despite this progress, significant gaps remain in defining causal relationships and specific microbial signatures that underlie vulnerability to trauma-related disorders. The present review therefore aims to provide an integrated synthesis of the patho-physiological mechanisms linking dysbiosis to trauma-related conditions, current pre-clinical and clinical evidence in PTSD, and translational implications for microbiota- and nutrition-based therapeutics. Importantly, rather than positioning PTSD as one condition among many within the gut–brain axis literature, this review conceptualizes PTSD as a paradigmatic model of stress-related systemic dysbiosis. PTSD uniquely integrates chronic stress exposure, neuroendocrine dysregulation, immune activation, and behavioral persistence, offering a clinically informative framework to interrogate gut–brain mechanisms across biological levels. Figure 1 illustrates the complex interactions between psychological trauma, the HPA axis, the gut microbiome, and central nervous system functioning. Exposure to trauma and chronic stress activates the HPA axis, with increased release of CRH and adrenocorticotropic hormone (ACTH), promoting neuroendocrine dysregulation and neuroinflammation. In parallel, stress-related immune activation contributes to gut dysbiosis, increased intestinal permeability (leaky gut), and altered production of microbial metabolites, including SCFAs and LPS. These gut-derived signals influence brain function through immune, endocrine, and neural pathways, including vagal signaling and neurotransmitter modulation, ultimately contributing to mood disorders, cognitive impairment, sleep disturbances, PTSD.

In contrast to recent reviews that primarily catalog microbiome associations or therapeutic candidates, the present work adopts a translational and stratification-oriented perspective. We critically evaluate preclinical versus clinical evidence, explicitly address causal limitations, and integrate nutritional psychiatry and lifestyle-based interventions within realistic clinical care pathways. By emphasizing patient heterogeneity, feasibility, and implementation barriers, this review aims to bridge mechanistic insights with clinically actionable frameworks, aligning with emerging precision psychiatry approaches.

## 2. Materials and Methods

### 2.1. Search Strategy and Selection Criteria

This narrative review integrated evidence from recent systematic and narrative reviews, together with selected primary studies identified through structured searches of PubMed, Scopus, and Web of Science, and supplemented by relevant publications known to the authors. Priority was given to articles addressing PTSD or trauma-exposed populations, with a specific focus on mechanistic investigations related to the GBA.

Key sources included conceptual and mechanistic frameworks on PTSD–microbiome interactions, meta-analyses examining inflammatory and microbial biomarkers in PTSD, longitudinal and observational studies assessing microbiome changes following trauma exposure, and experimental work, primarily preclinical, exploring dietary, microbial, or lifestyle-based interventions with potential relevance for stress resilience and symptom modulation [9,23]. A structured literature search was conducted across PubMed, Scopus, and Web of Science to identify relevant publications addressing the gut–brain axis in PTSD and trauma-related conditions. Searches covered the period from January 2011 to March 2025 and combined keywords related to trauma and stress-related disorders (“post-traumatic stress disorder”, “PTSD”, “trauma”, “stress-related disorders”) with microbiome- and gut–brain axis–related terms (“gut microbiota”, “microbiome”, “gut–brain axis”, “dysbiosis”, “intestinal permeability”, “psychobiotics”).

### 2.2. Inclusion and Exclusion Criteria and Time Frame

Eligible studies comprised human research (cross-sectional, longitudinal, or interventional) involving PTSD or trauma-exposed populations, as well as preclinical studies employing validated stress paradigms, germ-free or antibiotic-treated models, or fecal microbiota transplantation (FMT) to investigate mechanistic pathways linking stress, immunity, and microbiome alterations.

In addition, authoritative reviews providing mechanistic syntheses of GBA signaling, neuroendocrine stress responses, and immune–brain interactions were included to contextualize empirical findings [7,10,32]. The literature search covered studies published between 2011 and 2025, reflecting the period of rapid expansion in microbiome–mental health research.

Studies were excluded if they focused exclusively on non-gut microbial niches without a clear mechanistic link to PTSD-related biological processes, or if they consisted of non–peer-reviewed commentaries or isolated case reports. Given the narrative and translational focus of this review, study selection prioritized conceptual relevance, mechanistic insight, and clinical interpretability rather than exhaustive coverage. Priority was given to: human studies involving PTSD or trauma-exposed populations; preclinical studies employing validated stress paradigms relevant to PTSD pathophysiology; and reviews and meta-analyses providing integrative mechanistic frameworks. Studies were excluded if they lacked a clear link to gut–brain axis mechanisms or focused exclusively on non-gut microbial niches without translational relevance to PTSD.

**Figure 2:**
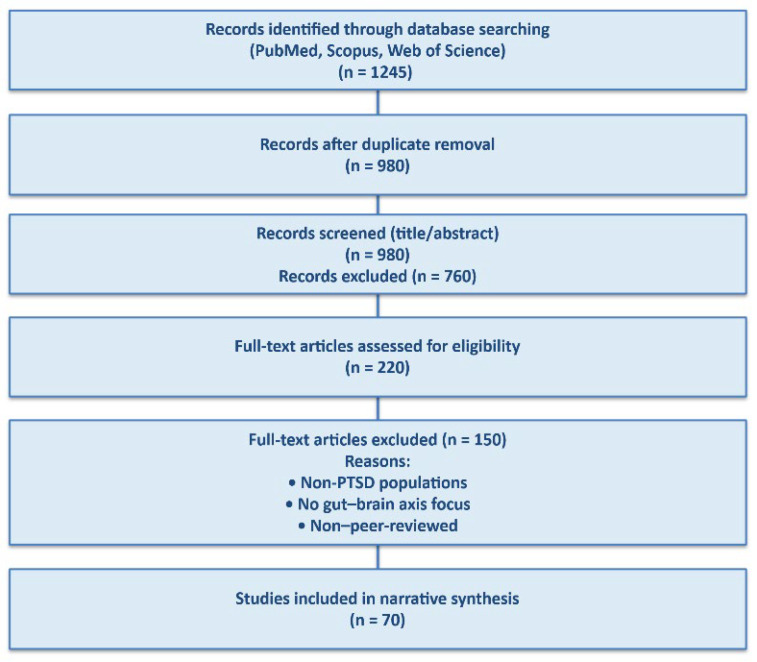
Flow-style summary of the literature search and study selection process.

A flow-style summary of the literature screening and selection process is provided in Figure 2 to enhance transparency and reproducibility. The figure illustrates database searching, screening, eligibility assessment, and final inclusion of studies in the narrative synthesis.

## 3. Pathophysiological Links Between Dysbiosis and Trauma

This section integrates immune, neuroendocrine, autonomic, and metabolic pathways to illustrate how gut dysbiosis may contribute to sustained stress-related biological dysregulation in PTSD, rather than to propose isolated mechanistic effects.

### 3.1. Dysbiosis, Gut Permeability, and Endotoxemia

Chronic stress profoundly alters the gut microbial composition, leading to loss of commensal diversity and increased abundance of pro-inflammatory taxa [9,16,22]. This imbalance compromises the intestinal epithelial barrier, weakening tight junction proteins such as claudins and occludin. The resulting increased permeability allows bacterial components such as lipopolysaccharides (LPS) to enter the systemic circulation, a phenomenon often referred to as “leaky gut” [16,22].

Both clinical and preclinical data converge on this barrier dysfunction as a key driver of low-grade endotoxemia and immune activation in trauma-related conditions. In animal models, repeated stress exposure induces LPS translocation, increased plasma cytokines, and behavioral alterations reminiscent of PTSD, while probiotic or SCFAs supplementation can restore barrier integrity [9,22]. Human studies report similar findings, showing altered gut permeability markers and inflammatory signatures in PTSD cohorts [9,16,22]. Similar mechanisms of oxidative stress, barrier dysfunction, and immune activation have been extensively described in irritable bowel syndrome, a condition frequently comorbid with stress-related psychiatric disorders [33]. The relevance of gut–brain signaling extends beyond psychiatric conditions, as alterations of the brain–gut axis have also been documented in systemic diseases such as gastrointestinal cancers, highlighting its broad pathophysiological relevance [34].

Collectively, these results support the concept that dysbiosis and gut barrier disruption initiate systemic inflammation, thereby linking psychological stress to peripheral and central immune activation.

### 3.2. Neuroendocrine Pathways: HPA Axis Dysregulation

PTSD is characterized by paradoxical HPA axis dysfunction, typically featuring elevated CRH levels but low circulating cortisol [3,9,20]. This “hypocortisolemic” profile contrasts with the hypercortisolism of acute stress and is thought to result from enhanced negative feedback sensitivity of glucocorticoid receptors in chronic trauma survivors [3].

Premorbid inflammatory tone, illustrated by elevated baseline CRP, has been shown to predict PTSD symptom severity after trauma exposure, underscoring the bidirectional crosstalk between immune activation and neuroendocrine set-points [3]. Dysbiosis and microbial metabolites may influence this axis by modulating cytokine signaling and tryptophan metabolism, both of which can alter glucocorticoid sensitivity [9,20].

HPA axis dysregulation has been linked to microbiota alterations, although directionality and causality remain to be established in humans, potentially perpetuating stress reactivity and the maintenance of PTSD symptomatology [3,9,20].

### 3.3. Neuroimmune Signaling and Microglial Activation

Inflammation constitutes another crucial interface between gut dysbiosis and brain function. PTSD and related disorders are often accompanied by elevated pro-inflammatory cytokines, including IL-6, TNF-α, and CRP, which have been implicated in mood and anxiety dysregulation [3,23,35]. Yet, meta-analyses show heterogeneous findings, with no consistent case–control mean differences across biomarkers, largely due to variability in sample size, comorbidities, and assay methodology [23,35]. Altered toll-like receptor (TLR) signaling, previously demonstrated in functional gastrointestinal disorders, provides a mechanistic framework linking microbial products to innate immune activation relevant also to PTSD [36,37].

In animal studies, dysbiosis-induced inflammation activates microglial cells in limbic and prefrontal areas, impairing neuroplasticity and promoting anxiety-like behaviors. The restoration of a healthy microbiome or supplementation with anti-inflammatory metabolites, such as butyrate, can attenuate microglial activation and normalize synaptic signaling [22,23].

Together, these observations suggest that gut-derived inflammatory cues and microglial activation represent a mechanistic bridge between peripheral immune dysfunction and central neural remodeling in PTSD.

### 3.4. Vagal/Autonomic Pathways

The vagus nerve constitutes a primary communication route between the gut and brain, transmitting microbial and immune signals through afferent fibers that project to the nucleus tractus solitarius and influence limbic circuitry [9,20]. Activation of vagal pathways triggers the cholinergic anti-inflammatory reflex, which inhibits cytokine release via α7 nicotinic receptors on macrophages [9].

Conversely, autonomic imbalance, characterized by sympathetic overdrive and reduced vagal tone, is a recurrent feature in PTSD, correlating with hyperarousal, sleep disturbance, and exaggerated startle response [9,20]. Gut-directed interventions such as vagal nerve stimulation, probiotics, and diet rich in fermentable fibers have shown promise in partially restoring autonomic regulation in stress-related conditions, although clinical data remain preliminary [9,20].

These findings underscore that the autonomic nervous system serves as both mediator and therapeutic target in the gut–brain connection of PTSD.

### 3.5. Microbial Metabolites and Neurotransmitters

Gut microbes synthesize and modulate numerous bioactive metabolites that influence neural and immune signaling. Among them, SCFAs, notably acetate, propionate, and butyrate, interact with G-protein-coupled receptors (FFAR2/3) and can cross the blood–brain barrier to a limited extent, where they modulate microglial maturation, histone deacetylase (HDAC) inhibition, and neuroinflammation [16,22].

In parallel, microbial enzymes regulate tryptophan availability, diverting metabolism toward the kynurenine pathway, which affects serotonergic and glutamatergic transmission [20]. Altered kynurenine/tryptophan ratios observed in stress models and PTSD patients suggest that dysbiosis-driven metabolic shifts may contribute to anxiety, anhedonia, and cognitive inflexibility [16,20,22].

Large-scale metagenomic analyses have demonstrated that the human gut microbiota harbors extensive neuroactive potential, including pathways involved in GABAergic and serotonergic signaling [38]. SCFAs, indoles, and microbial peptides collectively shape neuroplasticity, stress resilience, and affective tone [16,20,22].

Thus, microbial metabolites represent key chemical messengers through which the gut microbiota modulates brain function and behavior, bridging molecular, endocrine, and neural domains in PTSD.

Table 1 shows principal biological mechanisms linking dysbiosis, immune activation, and neuroendocrine dysregulation in PTSD.

## 4. Evidence from Preclinical Models

Preclinical models provide mechanistic and causal insights into gut–brain interactions under stress; however, their translational relevance to human PTSD remains partial and context-dependent.

### 4.1. Early-Life Stress and Microbiota Development

The early-life period represents a critical window for the establishment of immune homeostasis, neurodevelopment, and stress responsivity, all of which are strongly shaped by the gut microbiota [16,39,40]. Perturbations of microbial colonization during infancy—such as maternal separation, antibiotic exposure, early-life adversity, or prenatal stress—have been shown to induce long-lasting behavioral, endocrine, and immune alterations that resemble increased vulnerability to PTSD-like phenotypes later in life [39,40,41].

Preclinical studies demonstrate that early stress disrupts microbial diversity and delays the maturation of key commensal taxa involved in immune tolerance and neurodevelopment, including Lachnospiraceae and other butyrate-producing families [16,39]. These alterations coincide with exaggerated HPA axis reactivity, impaired glucocorticoid feedback, and microglial priming, suggesting that early microbiota maturation critically shapes stress resilience trajectories across the lifespan [40,41].

### 4.2. PTSD-like Phenotypes and Microbiota Alterations in Animals

Experimental paradigms of chronic stress, such as repeated social defeat stress, chronic unpredictable stress, or predator exposure, recapitulate key behavioral and biological features of PTSD, including hyperarousal, anxiety-like behavior, social avoidance, and neuroinflammatory activation [16,42,43]. Across these models, consistent alterations in gut microbiota composition have been observed, notably reduced abundance of SCFA-producing taxa (e.g., Lachnospiraceae, Ruminococcaceae), that has been associated with impaired stress resilience and altered neuroimmune signaling, and expansion of pro-inflammatory or stress-responsive bacterial groups [16].

Yadav et al. demonstrated that repeated social defeat stress induces mucosal immune activation, increased claudin-2 expression (indicative of tight-junction disruption), and elevated catecholaminergic signaling, directly linking stress neurobiology to gut barrier dysfunction [16]. Complementary studies show that stress-induced dysbiosis interacts with host immune and neuroplasticity-related pathways, reinforcing a bidirectional loop between microbial perturbations, inflammation, and maladaptive behavioral outcomes [42,44].

### 4.3. Germ-Free, Antibiotic, Colonization, and FMT (Fecal Microbiota Transplantation)

Germ-free (GF) animal models provide some of the most compelling evidence for a causal role of the microbiota in stress-related neurobehavioral regulation. GF mice exhibit exaggerated HPA axis responses to stress, increased anxiety-like behavior, altered social interactions, and reduced expression of neurotrophic factors such as brain-derived neurotrophic factor (BDNF) in limbic regions [5,45]. Importantly, colonization with a conventional microbiota early in life largely normalizes these phenotypes, whereas late colonization only partially restores neuroendocrine and behavioral balance, highlighting a developmental critical period for microbiota–brain interactions [5,41].

Antibiotic-induced microbiota depletion in adulthood similarly results in heightened stress sensitivity, immune dysregulation, and behavioral alterations, which can be reversed by probiotic administration or FMT from healthy donors [45,46]. Notably, FMT from stress-resilient or behaviorally healthy donors has been shown to transfer adaptive stress responses, whereas FMT from stressed donors can transmit anxiety-like and depressive phenotypes [47,48].

Specific strains within the Lactobacillus and Bifidobacterium genera have repeatedly demonstrated the capacity to attenuate stress-induced behavioral deficits, normalize HPA axis hyperreactivity, and modulate neurotransmitter systems including serotonin and γ-aminobutyric acid (GABA) [46,47,49]. Together, these findings establish a bidirectional and mechanistically grounded relationship between gut microbial composition and emotional regulation, providing strong preclinical support for microbiota-targeted interventions in trauma-related disorders.

### 4.4. Translational Considerations: Differences Between Animal Models and Human Trauma

While preclinical models have been instrumental in elucidating mechanistic links between stress, gut microbiota, and neurobiological outcomes, important differences must be acknowledged when translating these findings to human trauma-related disorders. Microbiome complexity differs substantially between laboratory animals and humans. Rodent microbiota is characterized by lower taxonomic diversity, standardized housing conditions, and controlled diets, which reduce environmental variability but limit ecological validity. In contrast, the human gut microbiome is shaped by highly heterogeneous factors including diet, geography, socioeconomic status, medication exposure, and cumulative life stress, resulting in markedly greater inter-individual variability [3,7].

Trauma exposure in animal models typically involves time-limited and experimentally controlled stress paradigms (e.g., social defeat, restraint stress, predator exposure), whereas human trauma is often characterized by chronicity, repetition, and ongoing psychosocial threat. Human PTSD frequently emerges in the context of prolonged adversity, moral injury, interpersonal violence, or cumulative developmental trauma, dimensions that are only partially captured by existing animal models.

Psychosocial and cognitive context represents a critical translational gap. Human PTSD is embedded within complex cognitive processes, including autobiographical memory, meaning-making, rumination, and social appraisal, as well as ongoing environmental contingencies such as safety, social support, and socioeconomic instability [37]. These dimensions interact with biological stress systems but cannot be directly modeled in animals.

Consequently, while animal studies provide strong internal validity and causal inference regarding microbiota–immune–neuroendocrine mechanisms, their findings should be interpreted as mechanistic hypotheses rather than direct proxies for human PTSD pathophysiology. Careful integration with longitudinal, well-phenotyped human studies is essential to define clinically meaningful microbiota targets and to avoid overgeneralization from preclinical evidence.

## 5. Clinical Evidence in PTSD and Trauma-Related Disorders

Clinical studies predominantly demonstrate associations rather than causality; nevertheless, converging patterns across microbiome composition, immune markers, and metabolic pathways support a biologically plausible role for gut–brain axis alterations in PTSD.

### 5.1. Cross-Sectional Microbiome Studies

Cross-sectional investigations of the gut microbiome in PTSD reveal consistent, though heterogeneous, alterations in microbial composition and diversity. Early exploratory studies first suggested that trauma exposure is associated with distinct gut microbial profiles compared with non-exposed controls [50], an observation later confirmed and expanded in larger cohorts.

In a recent multicenter case–control study, Zeamer et al. demonstrated that PTSD patients exhibited lower relative abundance of Actinobacteria, Lentisphaerae, and Verrucomicrobia, with marked reductions in Akkermansia and Bifidobacterium—genera critically involved in barrier integrity and mucosal immunoregulation [51]. Similar patterns were reported in independent clinical samples, where PTSD and trauma-exposed individuals showed reduced α-diversity, depletion of short-chain fatty acid (SCFA)–producing taxa such as Lachnospiraceae and Ruminococcaceae, and enrichment of potential pathobionts including Enterococcus and Escherichia/Shigella [29,35,52].

Systematic reviews synthesizing these findings highlight that, despite variability across cohorts, PTSD-related dysbiosis converges on the loss of beneficial commensals and expansion of pro-inflammatory species, paralleling microbial alterations described in other stress-related and inflammatory psychiatric conditions [21,35]. Collectively, these data suggest the presence of recurrent microbiome patterns associated with PTSD and trauma exposure, although such patterns appear to be strongly modulated by clinical heterogeneity, environmental context, and methodological factors rather than representing a single, uniform microbial signature.

### 5.2. Longitudinal/Prospective Studies

While cross-sectional designs limit causal inference, longitudinal studies have begun to clarify temporal relationships between trauma exposure, microbiome alterations, and symptom trajectories. The large-scale AURORA study, analyzed by Zeamer et al. (2023), reported that fecal metagenomic profiles collected approximately 45 days after trauma exposure explained up to 48% of the variance in PTSD symptom severity, 26% in depressive symptoms, and 44% in somatic complaints over a 2–12 weeks follow-up period [51].

Specific microbial taxa, including *Bifidobacterium adolescentis*, *B. longum*, *Flavonifractor plautii*, and *Ruminococcus gnavus*, were identified as predictors of worse psychiatric outcomes [50]. Pathway-level analyses further revealed reduced L-arginine biosynthesis and enhanced citrulline/ornithine cycling, consistent with prior reports of altered arginine bioavailability and nitric oxide signaling in PTSD [9,14].

Complementary Mendelian randomization and host–microbiome interaction studies suggest that genetic susceptibility interacts with microbial metabolic pathways regulating immune activation and neurotransmission, strengthening the case for a contributory role of gut microbiota in shaping post-trauma vulnerability and recovery trajectories [20]. Together, these findings support a temporal association between microbial metabolic pathways and PTSD symptom trajectories, consistent with, but not definitive evidence of, a contributory role of gut microbiota in post-trauma vulnerability and recovery.

### 5.3. Biomarkers: Permeability, Inflammatory Cytokines, and Metabolomics

Evidence from meta-analyses and pooled clinical studies provides a broader perspective on biological correlates of PTSD. Quantitative syntheses encompassing multiple cohorts have reported no consistent case–control differences across individual inflammatory markers such as IL-6, TNF-α, and CRP, alongside mixed microbiome signals, including non-significant pooled α-diversity measures and only partial depletion of SCFA-producing families [23,35].

As discussed by Petakh et al., this apparent inconsistency is largely attributable to methodological heterogeneity, including small sample sizes, variable trauma timing, comorbid depression or substance use, psychotropic medication exposure, and dietary confounders [23]. Nonetheless, converging trends toward low-grade systemic inflammation, altered immune signaling, and compromised intestinal permeability are repeatedly observed across diverse PTSD populations [3,28].

Emerging metabolomic studies further indicate dysregulation of amino acid pathways—particularly tryptophan–kynurenine and arginine metabolism—linking gut-derived metabolic shifts to immune activation and neuroendocrine dysfunction in trauma-related disorders [9,20]. These observations underscore the need for standardized biomarkers and longitudinal multimodal designs to disentangle causality within the gut–immune–brain interface.

### 5.4. Common Comorbidities

Beyond gut-focused investigations, accumulating evidence suggests that PTSD-related dysbiosis extends to extra-intestinal microbial niches, supporting a systemic rather than localized microbiome alteration. In a large community cohort, Malan-Müller et al. (2024) demonstrated that periodontal status and trauma-related symptom severity jointly shaped salivary microbial composition, with higher trauma burden associated with reduced Haemophilus sputorum and increased Prevotella histicola abundance [53].

Similarly, studies in trauma-exposed veterans have identified distinct oral microbiota signatures associated with PTSD symptom clusters, implicating shared inflammatory and neuroimmune pathways across microbial compartments [54]. Predicted metagenomic functions in these populations suggest altered tryptophan metabolism and serotonergic signaling, potentially contributing to sleep disturbances, fatigue, and increased cardiometabolic risk frequently observed in PTSD [53,54]. Metabolic comorbidities, including obesity, share overlapping gut–brain axis mechanisms with PTSD, suggesting partially convergent microbiota-driven pathways [55].

Together, these findings reinforce the concept that PTSD-related dysbiosis reflects a multisystem phenomenon, with interconnected gut, oral, immune, and metabolic networks influencing both mental and physical health outcomes in trauma survivors.

To address the heterogeneity and variable strength of available findings, Table 2 provides an integrative synthesis of the main gut–brain axis domains implicated in PTSD, summarizing the type and consistency of evidence and their current level of translational readiness. This framework is intended to support a critical interpretation of existing data and to guide the subsequent discussion of microbiota-targeted interventions.

To improve comparability and reproducibility in future PTSD–microbiome research, it is recommended to adopt established reporting and metadata standards. For human microbiome studies, authors should follow the STORMS checklist. For observational designs, core reporting should align with STROBE and, when molecular measures are central, the STROBE-ME extension. When animal data are used to motivate or interpret PTSD mechanisms, ARRIVE 2.0 should guide transparent reporting of experimental design and bias-reduction procedures. Finally, microbiome metadata should be standardized using Genomic Standards Consortium specifications (e.g., MIxS/MIMARKS) to ensure consistent documentation of sampling, processing, sequencing, and contextual variables.

## 6. Nutritional and Microbiota-Targeted Interventions

As summarized in Table 2, most microbiota-related mechanisms in PTSD are supported by converging but heterogeneous evidence, with varying degrees of translational readiness. Accordingly, microbiota-targeted interventions should currently be viewed as adjunctive and stratification-informed strategies rather than disorder-specific treatments.

### 6.1. Dietary Patterns (Mediterranean, Western, Plant-Forward)

Dietary patterns exert profound effects on the composition and metabolic activity of the gut microbiome, with downstream consequences for immune and neuroendocrine regulation. Mediterranean-style diets, rich in fruits, vegetables, whole grains, legumes, fermentable fibers, and polyunsaturated fatty acid (PUFA) precursors, are consistently associated with enhanced microbial diversity, increased short-chain fatty acid (SCFA) production, and attenuation of low-grade inflammation [13,56,57,58]. Mechanistically, these effects involve activation of anti-inflammatory signaling pathways, including peroxisome proliferator–activated receptor (PPAR) signaling, and improved intestinal barrier integrity [56,57].

Conversely, Western-style dietary patterns characterized by high intake of saturated fats, refined sugars, and ultra-processed foods promote dysbiosis, impaired tight-junction function, and systemic inflammatory activation, biological features overlapping with those described in PTSD and stress-related disorders [54,59,60]. Emerging evidence further suggests that plant-forward, fiber-rich dietary patterns may enhance stress resilience by supporting SCFA-producing taxa and stabilizing hypothalamic–pituitary–adrenal (HPA) axis activity [61,62].

### 6.2. Key Nutrients and Bioactives (Omega-3s, Polyphenols, Vitamins B/D, Minerals)

Among individual dietary components, omega-3 polyunsaturated fatty acids (*n*-3 PUFAs) are the most extensively investigated for their psychoneuroimmunological effects. Experimental and clinical studies demonstrate that *n*-3 PUFAs reduce pro-inflammatory cytokine production, enhance BDNF signaling, modulate fear-memory processing, and attenuate anxiety-related behaviors [6,56,59]. These effects appear partly mediated by microbiota-dependent mechanisms influencing lipid metabolism and immune tone [59].

Polyphenols, B vitamins, vitamin D, and trace minerals further contribute to gut–brain axis modulation by shaping microbial composition, enhancing antioxidant defenses, and regulating inflammatory cascades [21,63]. Collectively, these bioactives act on convergent pathways involved in oxidative stress, immune activation, and neuroendocrine dysregulation, all of which are implicated in trauma-related psychopathology [6,7,63].

### 6.3. Prebiotics and Dietary Fiber

Prebiotics and fermentable fibers, including inulin, β-glucans, fructooligosaccharides, and resistant starches, selectively stimulate the growth of beneficial commensals, leading to increased SCFA production and reinforcement of epithelial barrier integrity [9,22,60]. SCFAs such as butyrate and propionate enhance tight-junction protein expression, suppress endotoxin translocation, and modulate peripheral immune signaling [60,64].

By dampening systemic inflammation and indirectly influencing microglial activation and HPA axis responsiveness, prebiotic supplementation may foster a biological milieu conducive to stress resilience and recovery [25,64]. Thanks to these and other mechanisms, dietary and probiotic interventions have shown potential benefits on stress-related symptoms and biological markers, but evidence in PTSD remains preliminary [22,61].

### 6.4. Probiotics and Psychobiotics

A growing body of preclinical and early-phase clinical research supports the concept of “psychobiotics”, probiotic strains capable of modulating emotional behavior and stress physiology. Specific Lactobacillus and Bifidobacterium strains have been shown to reduce anxiety- and depression-like behaviors, normalize exaggerated HPA axis responses, and decrease circulating inflammatory cytokines in stress models [31,49,65]. Emerging hypotheses also suggest that the therapeutic effects of psychedelics such as psilocybin may partly involve gut–brain axis modulation, including microbiota-driven effects on stress responsivity and neuroplasticity [66].

Human studies, though limited in PTSD populations, suggest beneficial effects on mood, stress perception, and immune markers, albeit with considerable heterogeneity in strain selection, dosing, and treatment duration [67,68]. These limitations underscore the need for standardized, mechanistically informed psychobiotic protocols tailored to trauma-related disorders [65,68].

### 6.5. Postbiotics and Short-Chain Fatty Acids (SCFAs)-Focused Strategies

Beyond live microorganisms, postbiotics, non-viable bacterial products and metabolites, are emerging as promising therapeutic tools. Butyrate, in particular, exerts pleiotropic effects on gut–brain communication by enhancing tight-junction protein expression (e.g., ZO-1, occludin, claudins), inhibiting histone deacetylases (HDACs), and regulating microglial maturation and activation [22,24].

These epigenetic and immunomodulatory actions provide a mechanistic rationale for SCFA-focused strategies aimed at restoring barrier integrity and reducing neuroinflammation in stress-related conditions [24,32]. Although PTSD-specific clinical trials are lacking, convergent preclinical evidence supports further translational exploration of postbiotic-based interventions [22,32].

Mechanistic frameworks across the microbiome–immune–neuronal interface support a plausible role for postbiotics and SCFAs in shaping systemic inflammation, blood–brain barrier integrity, and downstream neuroimmune signaling. However, translating these pathways into PTSD therapeutics remains early and indirect. Most human interventions currently modulate SCFAs biology upstream (e.g., via diet, prebiotics/probiotics, or broader microbiome-targeted strategies) rather than delivering defined SCFAs as pharmacologic agents, and definitive clinical evidence linking SCFAs target engagement to PTSD symptom improvement is still limited. In this context, SCFAs are best positioned as mechanistically grounded candidate mediators and biomarkers within a broader gut–immune–brain framework, rather than as established stand-alone treatments.

Several barriers complicate development of “SCFAs-based therapies” for PTSD. SCFAs are endogenous metabolites with challenging pharmacokinetics and site-of-action constraints: achieving reproducible, sustained colonic exposure (and downstream systemic/central effects) often requires specialized formulations, creating manufacturing, stability, and dose-standardization hurdles. In addition, eterogeneity in PTSD (trauma type, chronicity, comorbidity, diet, medications, and baseline microbiome function) increases variance and may obscure signal unless trials stratify participants using prespecified microbiome/SCFAs profiles and immune phenotypes. The clinical evidence base for microbiome–immune–neuronal mechanisms has not fully resolved causality in humans, and the review literature emphasizes that cause–effect relationships demonstrated in preclinical settings frequently remain difficult to confirm in clinical populations. Finally, regulatory pathways depend on product classification and claims: “postbiotics” (as defined by consensus frameworks) may be feasible as health products, but positioning them as treatments for psychiatric disorders typically raises drug-level requirements for quality, consistency, and reproducible bioactivity.

Accordingly, current evidence supports SCFAs/postbiotics as biologically plausible targets within the gut–immune–brain axis, but the field needs adequately powered, well-phenotyped PTSD trials with standardized diagnostics, prespecified SCFAs and immune endpoints, rigorous control of major confounders (dietary intake, antibiotics, psychotropics), and transparent reporting to narrow the translational gap [69].

### 6.6. Fermented Foods

Fermented foods such as yogurt, kefir, kimchi, sauerkraut, and kombucha deliver live microbes alongside bioactive compounds that may influence gut–brain signaling. Observational and interventional studies in non-PTSD populations report associations between fermented food consumption, reduced perceived stress, improved affective regulation, and favorable cortisol profiles [31,68].

While these findings are preliminary and indirect, they suggest that fermented foods may represent a low-risk, culturally adaptable strategy to support microbial diversity and emotional well-being in trauma-exposed individuals [68]. Controlled trials in PTSD populations remain an important unmet need.

### 6.7. Fecal Microbiota Transplantation (FMT)

FMT provides the most direct method for restoring microbial diversity and community structure. Although no PTSD-specific FMT trials have been conducted, evidence from depression, autism spectrum disorder, and stress-related animal models demonstrates reversible effects on behavior, immune activation, and neurochemical signaling following microbiota transfer [45,48,70].

FMT has demonstrated microbiota-modulating and behavioral effects in experimental settings, but its role in PTSD remains exploratory and investigational. These findings support the conceptual relevance of FMT for trauma-related disorders while simultaneously highlighting critical challenges related to safety, donor selection, engraftment durability, and ethical considerations [48,71]. Carefully designed exploratory trials are required before clinical translation can be considered [71].

### 6.8. Multidomain Lifestyle Interventions: Diet, Exercise and Sleep

**Figure 3:**
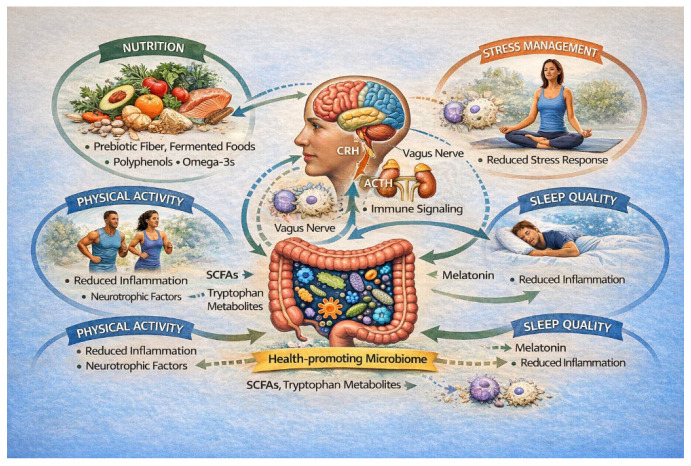
Effects of lifestyle interventions on gut–brain-immune axis. Note. Mediterranean diet, prebiotics, and healthy lifestyle factors (exercise and sleep) modulate the gut–brain–immune axis. Abbreviations: ACTH = adrenocorticotropic hormone; CRH = corticotropin-releasing hormone; SCFAs = short-chain fatty acids.

Multimodal lifestyle interventions integrating dietary modification, physical activity, and sleep regulation may exert synergistic effects across microbial, immune, autonomic, and neuroendocrine systems [1,25,72]. Physical exercise and mind–body practices such as yoga have been shown to increase microbial diversity, enhance SCFAs production, and upregulate BDNF signaling, while simultaneously reducing inflammatory tone [25,72]. Psychotherapeutic approaches, including cognitive-behavioral therapy (CBT) and mindfulness-based interventions, further modulate immune pathways such as TLR4/NF-κB signaling, suggesting convergence between psychological and biological mechanisms of recovery [3,72]. Figure 3 provides a simplified overview of how key lifestyle interventions (nutrition, physical activity, stress management, and sleep quality) modulate the gut–brain–immune axis. Healthy dietary patterns, such as Mediterranean diet, rich in prebiotic fibers, polyphenols, and omega-3 fatty acids, promote a health-supporting gut microbiome and the production of beneficial microbial metabolites. Physical activity and adequate sleep contribute to reduced systemic inflammation and improved neuroendocrine and immune regulation, while stress-management practices attenuate HPA axis activation and vagal dysregulation. Through integrated neural, endocrine, and immune pathways, these lifestyle factors collectively support brain function, immune homeostasis, and psychological resilience.

### 6.9. Safety, Tolerability, and Interactions with Psychotropics

The interaction between microbiota-targeted interventions and psychotropic medications represents a critical but underexplored clinical issue. Many antidepressants and antipsychotics exhibit antimicrobial activity that can alter gut microbial composition, potentially influencing metabolic side effects and treatment response [10,19].

For example, antipsychotic-associated weight gain and metabolic dysregulation appear modifiable through adjunctive probiotic strategies, implicating microbiota-mediated mechanisms in psychotropic tolerability [19]. Moreover, microbial modulation of opioid receptor signaling and reward pathways suggests a broader role of the gut microbiome in pharmacodynamics and stress-related vulnerability [4,73].

Careful monitoring of drug–microbiota interactions will be essential to ensure the safety, personalization, and clinical effectiveness of microbiota-based adjunctive therapies in PTSD and related disorders [10,73].

Overall, most microbiota-targeted interventions in PTSD remain supported primarily by preclinical and pilot-level evidence. At present, these strategies should be considered adjunctive and hypothesis-generating rather than disorder-specific treatments, pending adequately powered randomized controlled trials. To facilitate a critical interpretation of the intervention literature and to avoid overinterpretation of preliminary findings, Table 3 summarizes microbiota-targeted interventions according to a hierarchical level of evidence, ranging from preclinical studies to pilot clinical investigations and randomized controlled trials.

## 7. Moderators and Special Populations

### 7.1. Sex/Gender Differences

Sex hormones exert pleiotropic effects on immune regulation, epithelial barrier function, and gut microbial ecology, thereby shaping individual vulnerability to stress-related disorders [3,9,40]. Estrogen and progesterone modulate intestinal permeability, mucosal immunity, and microbial diversity, while also interacting with glucocorticoid signaling and neuroimmune pathways relevant to PTSD pathophysiology [3,40].

Epidemiological data consistently indicate a higher lifetime prevalence of PTSD in women, and preclinical studies reveal sex-specific microbiota and neuroimmune responses to chronic stress exposure [16,40]. Female rodents exposed to repeated stress paradigms show greater reductions in SCFA-producing taxa, heightened microglial activation, and exaggerated inflammatory signaling compared to males, suggesting sexually dimorphic microbiota–brain interactions [16,40,42]. Mechanistically, sex-related differences converge on gut–brain axis pathways described in Section 3 and Section 4. Sex hormones modulate intestinal barrier integrity, SCFA production, and immune signaling, thereby influencing HPA axis reactivity and microglial activation under stress. These sexually dimorphic microbiota–immune interactions may contribute to differential stress resilience and PTSD vulnerability across sexes.

These findings underscore the necessity for future PTSD–microbiome studies to stratify analyses by sex, incorporate hormonal status, and explore microbiota–endocrine interactions as potential mediators of resilience or vulnerability [3,9,16].

### 7.2. Early Life/Childhood/Adolescence

Early life constitutes a sensitive developmental window during which gut microbiota, immune calibration, and neuroendocrine systems co-mature. Exposure to early-life stress, antibiotic use, and adverse nutritional environments can induce persistent microbiome–immune alterations that increase susceptibility to PTSD and related disorders later in life [16,21,39].

Epigenetic mechanisms have been implicated in the intergenerational transmission of trauma-related vulnerability, potentially interacting with early-life microbiota programming [74]. Evidence from prenatal and perinatal cohorts supports this developmental framework: Naudé et al. (2020) demonstrated that maternal psychological distress during pregnancy is associated with significant alterations in both maternal and infant gut microbiota, including reduced Bifidobacterium abundance and enrichment of pro-inflammatory taxa, potentially influencing immune and metabolic programming before birth [39].

Experimental models further indicate that neonatal stress or early antibiotic exposure disrupt microbial colonization trajectories, leading to long-term HPA axis hyperreactivity, immune dysregulation, and anxiety-like behavior [16,41]. Adolescence represents an additional period of vulnerability, characterized by hormonal shifts, synaptic remodeling, and evolving dietary patterns that continue to shape gut–brain axis function [21,41].

Collectively, these findings highlight the importance of longitudinal developmental studies aimed at identifying early microbial predictors of trauma vulnerability and evaluating microbiota-targeted preventive strategies in pediatric and perinatal populations [16,21,39,41].

### 7.3. Socioeconomic, Cultural, and Dietary Context

Socioeconomic adversity, displacement, and cultural dietary patterns profoundly shape both microbiota composition and stress biology [75]. Nutritional access, food diversity, and environmental exposures differ across populations, affecting immune tone and gut microbial resilience [13,54].

Research on displaced and refugee populations highlights persistent psychosocial stressors (e.g., instability, malnutrition, crowding) as drivers of chronic inflammation and microbial imbalance, but also identifies resilience factors, such as community cohesion and traditional diets rich in plant fibers and fermented foods [75]. According to the “old friends” hypothesis, reduced exposure to immunoregulatory microbes may impair stress resilience and promote chronic inflammatory states relevant to trauma-related disorders [76].

Integrating socioeconomic and cultural moderators into PTSD–microbiome models is essential for developing GBA-informed interventions that are both equitable and contextually adapted [13,54,75]. From a mechanistic perspective, socioeconomic adversity likely operates through pathways outlined in Section 3 and Section 4, including reduced microbial diversity, impaired SCFA production, and chronic low-grade inflammation. These alterations may amplify stress-related immune activation and neuroendocrine dysregulation, thereby modulating PTSD risk and symptom persistence.

### 7.4. Medication Use and Microbiome Interactions

Pharmacotherapy remains central to PTSD management, yet antidepressants and antipsychotics exert antimicrobial effects that reshape microbial communities and metabolic outcomes [10,19]. Long-term exposure to psychotropics can influence microbial diversity, intestinal permeability, and weight regulation, sometimes mediating adverse metabolic profiles [19].

Accordingly, medication history should be modeled as both a confounder and a potential moderator in microbiome–PTSD analyses, as drug-related microbial shifts may alter inflammatory tone, treatment response, and overall homeostasis. Future studies should systematically evaluate drug–microbiota interactions using pharmacomicrobiomic frameworks to optimize personalized, gut-informed psychiatric care [10,19].

## 8. Clinical Translation

The added value of this review lies not in proposing a single PTSD-specific microbiome signature, but in framing gut microbiota alterations as modulators of stress vulnerability and recovery across heterogeneous trauma-related phenotypes. This shift from condition-centered to systems-oriented interpretation may help reconcile inconsistencies across studies and guide more feasible, patient-centered translational strategies. Importantly, translation of gut–brain–microbiota findings into clinical PTSD care is constrained by substantial interindividual variability, including sex, developmental timing of trauma exposure, psychiatric comorbidities, medication use, and socioeconomic context. These sources of heterogeneity likely contribute to inconsistent findings across clinical studies and variable responsiveness to microbiota-targeted interventions. Accordingly, translational strategies must prioritize stratification, feasibility, and real-world applicability rather than uniform treatment models.

### 8.1. Practical Guidance for Clinicians

The clinical relevance of the gut–brain–microbiota framework in PTSD is increasingly supported by converging evidence from psychoneuroimmunology, neurogastroenterology, and nutritional psychiatry, and can begin to inform pragmatic clinical practices beyond experimental settings [59,63,77].

Routine clinical assessment in PTSD should extend beyond core psychiatric symptoms to include diet quality, gastrointestinal function, sleep patterns, physical activity, and oral/periodontal health, all of which interact with immune and microbial homeostasis and may contribute to symptom persistence or treatment resistance [31,57,62]. However, time constraints, limited nutritional training among mental health clinicians, and variability in access to multidisciplinary care represent practical barriers to systematic implementation, underscoring the need for simplified screening tools and referral pathways.

Dietary counseling represents a low-risk, scalable intervention. Mediterranean-style dietary patterns, rich in dietary fiber, omega-3 fatty acids, polyphenols, and fermented foods, have been consistently associated with increased microbial diversity, enhanced SCFAs production, and attenuation of systemic inflammation, mechanisms directly relevant to stress-related neuroendocrine and immune dysregulation [58,65,67].

Although microbiota-informed interventions are not yet incorporated into formal PTSD guidelines, interdisciplinary collaboration between psychiatrists, nutrition professionals, and primary care clinicians may facilitate early implementation of evidence-informed lifestyle counseling within trauma-focused care pathways [59,77,78].

### 8.2. Candidate Clinical Algorithms and Care Pathways

A stepwise and integrative clinical framework may offer a pragmatic approach for translating microbiota science into PTSD care while respecting current evidentiary limits. First of all, clinicians should optimize established first-line treatments, including trauma-focused psychotherapies and guideline-concordant pharmacotherapy. Second, modifiable lifestyle factors (diet quality, sleep hygiene, physical inactivity, and substance use) should be systematically addressed, given their established effects on inflammation, autonomic balance, and microbial ecology [31,63,79].

Third, microbiota-targeted strategies may be considered as adjunctive measures, including increased intake of fermentable fibers, cautious use of strain-specific probiotics, and regular consumption of fermented foods with documented safety profiles. Their clinical scalability varies substantially, as probiotics and postbiotics differ in strain specificity, regulatory oversight, cost, and patient adherence, while more advanced approaches such as FMT currently remain confined to experimental or highly specialized settings [57,67,80]. Finally, in highly comorbid or treatment-refractory cases, referral to specialized or research-based protocols, such as metabolomic profiling, inflammatory phenotyping, or exploratory FMT studies, may be appropriate within controlled clinical or experimental contexts [59,81].

This staged approach is consistent with emerging precision psychiatry and systems-medicine models, which emphasize the integration of psychopharmacological, behavioral, immune, and metabolic domains to address the biological heterogeneity of trauma-related disorders [63,68,77].

### 8.3. Patient-Centered Outcomes and Feasibility

Effective clinical translation of GBA research requires prioritization of patient-centered outcomes that extend beyond symptom reduction alone. Fatigue, sleep quality, gastrointestinal comfort, emotional regulation, cognitive clarity, and functional recovery represent outcomes of high relevance to patients and are closely linked to immune and metabolic health [31,62,70].

Interventions should emphasize feasibility and shared decision-making, particularly when discussing dietary modifications, supplements, or probiotics, acknowledging the variability of individual responses and the still-evolving evidence base [57,67]. Cultural context, socioeconomic constraints, and food accessibility must be explicitly considered, especially in vulnerable populations exposed to chronic stress, displacement, or nutritional insecurity, where microbiota resilience may already be compromised [58,82].

The goal of clinical translation is not to replace established PTSD treatments, but to develop sustainable, adjunctive, and patient-centered care models that integrate dietary, psychological, and pharmacological strategies, while systematically monitoring safety, tolerability, adherence, and potential interactions with psychotropic medications [60,79]. Implementation of such models will require longitudinal monitoring, clinician education, and integration with existing care infrastructures, rather than parallel or experimental-only pathways.

While rodent stress models offer strong internal validity, their external validity for human PTSD is necessarily bounded. PTSD is a diagnosis anchored to symptom clusters, duration criteria, and functional impairment, whereas animal models operationalize stress-related phenotypes through behavioral proxies (e.g., startle, freezing, avoidance, anhedonia-like measures). This creates a construct-validity gap: similar biological changes (e.g., HPA-axis alterations, inflammatory shifts) may reflect general stress adaptation rather than PTSD-specific pathophysiology. In addition, human PTSD is shaped by cognitive processes (autobiographical memory, rumination, moral injury), interpersonal factors, and ongoing threat or safety signals, which are difficult to model in rodents. Differences in developmental timing, sex-specific effects, prior stress history, housing conditions, and strain further influence outcomes and can reduce reproducibility across paradigms. Consequently, our conclusions should be viewed as mechanistic hypotheses that require confirmation in well-characterized human cohorts (ideally with longitudinal designs, standardized PTSD diagnostic assessments, and control for trauma type and comorbidities), rather than as direct evidence of PTSD pathogenesis.

Table 4 provides a structured overview of the main microbiota-related mechanisms and outcomes reported across the included studies, integrating preclinical and clinical evidence.

## 9. Conclusions, Research Gaps and Future Directions

The GBA represents a promising and unifying framework for understanding the pathophysiology of PTSD and related trauma-spectrum conditions. Evidence from preclinical and early clinical studies supports a bidirectional relationship between stress-induced dysbiosis, immune–neuroendocrine dysregulation, and behavioral outcomes, suggesting that gut microbiota alterations are associated with PTSD symptom severity and persistence, and may contribute to stress-related biological dysregulation [9,22,23,51,57]. Despite rapid advances, current findings remain heterogeneous. Heterogeneity across PTSD populations, including differences in trauma type, chronicity, symptom profiles, comorbidities, and pharmacological treatment, likely contributes to the variability and partial non-replication observed across microbiome and immune studies. Future research should prioritize standardized, longitudinal, and multi-site designs with careful control of diet, medication, and lifestyle factors, integrating microbiome, immune, metabolic, and neuroendocrine measures [49,57,65]. Key mechanistic targets include intestinal barrier integrity, short-chain fatty acid signaling, tryptophan–kynurenine metabolism, arginine bioavailability, and autonomic/vagal modulation, which collectively shape stress reactivity and resilience [7,20,22,23]. From a translational perspective, nutritional, prebiotic, probiotic, and lifestyle-based interventions represent promising adjunctive strategies for PTSD prevention and treatment, warranting patient-centered clinical trials focused on functional outcomes such as mood, sleep, cognition, and gastrointestinal symptoms [30,54,63,77]. In conclusion, continued longitudinal and mechanistic research will be essential to validate current findings, refine causal models, and guide clinical translation into integrative, precision-oriented trauma care.

## Figures and Tables

**Table 1:** Principal biological mechanisms linking dysbiosis, immune activation, and neuroendocrine dysregulation in PTSD.

Pathway	Mechanistic Description	Representative Findings/Biomarkers	References
Barrier Dysfunction	Stress-induced tight-junction loss (“leaky gut”) → LPS translocation	↑ Zonulin, LPS, CRP	[9,16,22]
Neuroimmune Activation	Cytokine release and microglial activation	↑ IL-6, TNF-α; altered SCFA profile	[3,23,35]
Neuroendocrine Axis	HPA dysregulation, CRH ↑, cortisol ↓	Hypocortisolism, elevated CRP	[3,9,20]
Microbial Metabolites	Altered SCFAs, tryptophan/kynurenine, arginine	↓ Butyrate, altered Trp/Kyn ratio	[16,21,22]

Abbreviations: PTSD, post-traumatic stress disorder; LPS, lipopolysaccharide; SCFAs, short-chain fatty acids; HPA axis, hypothalamic–pituitary–adrenal axis; CRH, corticotropin-releasing hormone; IL-6, interleukin-6; TNF-α, tumor necrosis factor-alpha; CRP, C-reactive protein; Trp/Kyn, tryptophan/kynurenine pathway; ↑ increase; ↓ decrease.

**Table 2:** Integrative synthesis of gut–brain axis mechanisms in PTSD: evidence consistency and translational relevance.

Gut–Brain Axis Domain	Key Mechanism(s)	Main Evidence Source	Consistency of Findings	Translational Readiness	Key Limitations
Microbiota composition	Reduced α-diversity; depletion of SCFA-producing taxa (e.g., *Lachnospiraceae*, *Ruminococcaceae*); enrichment of pro-inflammatory taxa	Animal models; cross-sectional and longitudinal human studies	Moderate (heterogeneous across cohorts)	Early	High inter-individual variability; strong influence of diet, medication, and comorbidities
Intestinal barrier function	Increased gut permeability; LPS translocation; altered tight-junction proteins	Robust animal data; limited human biomarker studies	Moderate (stronger in preclinical models)	Early	Scarcity of validated permeability biomarkers in PTSD; indirect clinical measures
Immune and inflammatory signaling	Low-grade systemic inflammation; altered cytokine profiles (e.g., IL-6, TNF-α, CRP)	Meta-analyses; observational clinical studies	Low–Moderate	Indirect	Substantial heterogeneity; confounding by obesity, smoking, and psychotropic use
Neuroendocrine regulation (HPA axis)	Hypocortisolism; enhanced CRH signaling; altered glucocorticoid feedback	Human observational and longitudinal studies	Moderate	Established (pathophysiology)	Limited specificity to microbiota-driven mechanisms
Microbial metabolites	Altered SCFAs availability; disrupted tryptophan–kynurenine and arginine pathways	Preclinical studies; emerging human metabolomics	Emerging	Early	Limited clinical validation; lack of standardized metabolomic panels
Autonomic and vagal pathways	Reduced vagal tone; impaired cholinergic anti-inflammatory reflex	Preclinical studies; indirect human evidence	Emerging	Early	Predominantly associative clinical data
Microbiota-targeted interventions	Diet, prebiotics, probiotics, postbiotics, lifestyle interventions	Animal studies; pilot and small clinical trials	Variable	Emerging	Small sample sizes; strain- and context-specific effects; limited PTSD-specific RCTs

Abbreviations: SCFAs, short-chain fatty acids; LPS, lipopolysaccharide; HPA axis, hypothalamic–pituitary–adrenal axis; CRH, corticotropin-releasing hormone; IL-6, interleukin-6; TNF-α, tumor necrosis factor-α; CRP, C-reactive protein.

**Table 3:** Evidence hierarchy for microbiota-targeted interventions in PTSD.

Intervention Category	Preclinical Evidence	Pilot Clinical Data	RCT-Level Evidence	Current Interpretation
Dietary patterns (Mediterranean, fiber-rich)	Strong	Moderate	Limited (indirect)	Adjunctive, low-risk
Probiotics/Psychobiotics	Strong (strain-specific)	Preliminary	Absent (PTSD-specific)	Experimental adjunct
Prebiotics/Postbiotics	Strong (mechanistic)	Emerging	Absent	Mechanistic candidates
Lifestyle interventions (exercise, sleep)	Moderate	Moderate–Strong	Indirect	Clinically actionable
Fecal microbiota transplantation (FMT)	Strong (animal models)	Very limited	Absent	Experimental only

**Table 4:** Main Microbiota-related mechanisms and outcomes in the included studies.

Intervention Type	Mechanism of Action	Evidence Level	References
Dietary (Mediterranean/fiber-rich)	Promotes SCFAs, reduces inflammation	Moderate (observational, small trials)	[12,53,62,75]
Probiotics (Lactobacillus/Bifidobacterium)	Modulate cytokines, HPA activity	Preliminary (animal, pilot human)	[9,22,54]
Prebiotics/Postbiotics	Enhance barrier function, modulate SCFAs	Emerging (mechanistic studies)	[16,22,54]
Lifestyle (exercise, sleep, CBT)	Regulates autonomic and immune tone	Robust (indirect evidence)	[1,63]
FMT	Restores microbiota composition	Experimental (research phase)	[9,54]

Abbreviations: HPA, hypothalamic–pituitary–adrenal; SCFAs, short-chain fatty acids; CBT, cognitive behavioral therapy; FMT, fecal microbiota transplantation.

## Data Availability

No new data were created or analyzed in this study. Data sharing is not applicable to this article.
